# Clinical Variant of Ablepharon Macrostomia Syndrome

**DOI:** 10.1155/2011/593045

**Published:** 2012-01-05

**Authors:** Jose Larumbe, Patricia Villalta, Ines Velez

**Affiliations:** ^1^Pediatric Dentistry, Nova Southeastern University, 3200 S University Drive, Fort Lauderdale, FL 33328, USA; ^2^Oral and Maxillofacial Pathology, Nova Southeastern University, 3200 S University Drive, Fort Lauderdale, FL 33328, USA

## Abstract

Ablepharon syndrome is an extremely rare genetic problem that causes severe craniofacial deformities and numerous other abnormalities of the face, genitalia, and skin. The literature regarding this condition is scarce. We present a case of this syndrome with dental manifestations, not reported before, and discuss its characteristics in order to increase the knowledge of this condition among the dental profession.

## 1. Introduction

Ablepharon macrostomia syndrome (AMS) is an extremely rare and disfiguring condition, characterized by numerous signs and symptoms. The literature about this disease is sparse with only a few cases reported. AMS affects several systems and tissues of the body, such as skin, genitalia, craniofacial structures, and fingers. AMS has been reported to be an autosomal recessive entity, and it has been suggested that the gene maps to 18q [1]. Cruz et al. [2] described affected siblings, and Ferraz [3] reported a familial case which affected father and son.

The word Ablepharon means absence of eyelids. In 1977 McCarthy and West [4] reported two cases of a rare syndrome characterized by numerous manifestations such as triangular facies, hypertelorism, sparse thin hair, absence of eyelids, eyebrows, and eyelashes, eye abnormalities, and dry skin. They also found rudimentary pinnae, low set ears with collapsed canals and hearing loss, small nose with triangular nostrils, thin lips, wide “fish-like” mouth (macrostomia), and redundant skin folds. 

Later, Price et al. [5], Hornblass and Reifler [6], and Jackson et al. [7] reported cases with similar manifestations and added other signs/symptoms such as hypoplastic nipples and webbed fingers associated with delayed development of language and in some cases mental retardation. A few more cases had been reported since then [8]. There are descriptions of more characteristics pertaining to the same condition, such as ambiguous genitalia with posteriorly placed micropenis and absence of scrotum, ventral hernia, alterations of the abdominal wall, lack of subcutaneous tissue, and absence of zygomatic arches [8]. 

Barber-Say syndrome which presents similar manifestations should be considered within the differential diagnosis. The latest is also characterized by hypertrichosis [9].

## 2. Case Report

A 4-year-old African American male with severe craniofacial deformities presented with his parents to the Pediatric Dentistry Clinic at Nova Southeastern University, for a consultation. Medical history disclosed a 38-week normal pregnancy and no suspected problems before birth. Parents were no related, no teratogens were identified during pregnancy, and there is no family history of birth defects. No signs of abuse or neglect were noted during the consultation.

At birth, however, his doctors were confronted with a child with absent eyebrows, abnormal face appearance, underdeveloped ears, and dry, thickened, scaly skin (congenital ichthyosis). Hematological and biochemical profiles were within normal limits.

**Figure 1:**
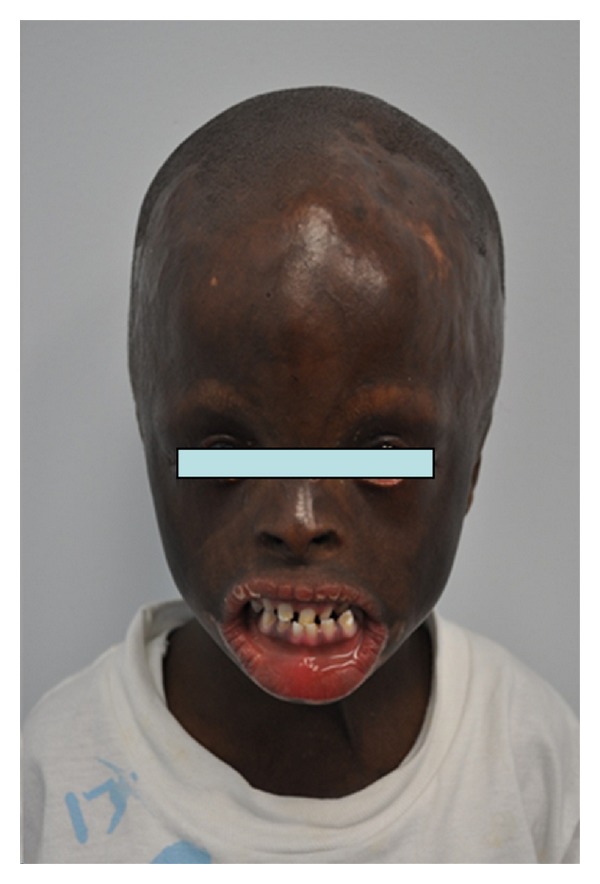
Marked craniofacial deformities.

**Figure 2:**
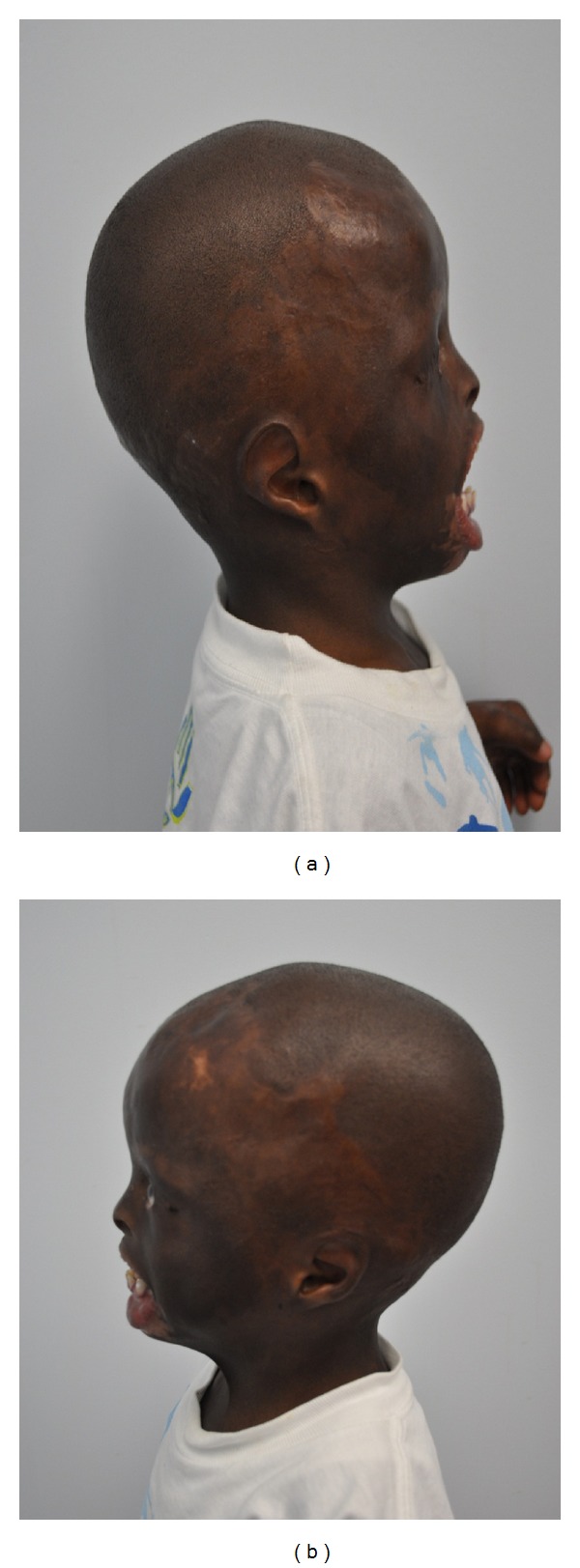
Hypoplastic ears fused to head, underdeveloped nose, and macrostomia.

**Figure 3:**
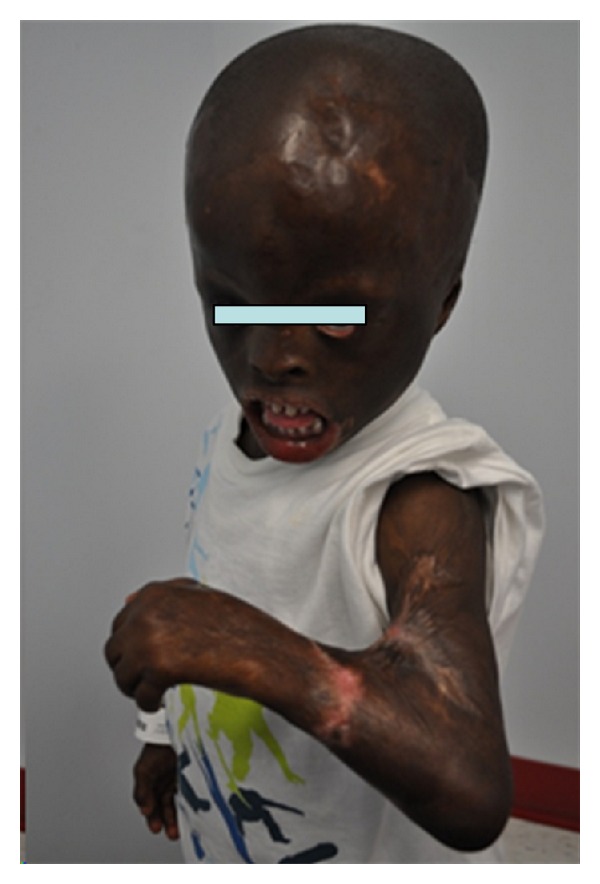
Severe contracture of the left elbow.

Extraoral examination at 4 years of age revealed marked craniofacial deformities including prominent triangular head, extremely large forehead, alopecia, hypoplastic zygomatic arches, constantly open eyes, due to the absence of eyelids, requiring the continuous need for eye drops, rudimentary eyelids, no eyelashes nor eyebrows (Figure 1). Hypoplastic ears fused to head, underdeveloped nose, and macrostomia (Figures 2(a) and 2(b)). Severe scarring of the skin was a prominent feature especially on the lower lip, the back, the chest, the wrists, and the palms. He showed limited finger motion, as well as severe contracture of the left elbow, due to the tightness of the scarred skin (Figure 3).

Neurologically, the patient presented good responses and normal mental function; he was able to walk and talk. The patient developed into a personable tough guy who understands his condition. He has some friends and realizes that people might stare at him.

Intraoral examination revealed complete primary dentition with enamel hypoplasia (not reported before) and stains (Table 1). No cavities were seen.

Periodontal Examination revealed gingival tissue within normal limits, pink and stipple, no signs of inflammation. 

Occlusion: Molars: R/L: class I Canine: R: class III. L: b/b.

**Figure 4:**
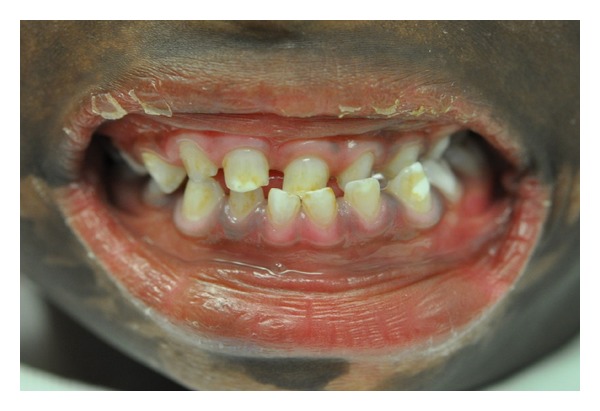
Generalized.

Middle line shifted toward left, protrusion of left lower anterior teeth and inverted anterior bite (Figure 4).

The treatment of patients with this syndrome needs extreme dedication and devotion from the family. A team composed of a pediatrician, a pediatric dentist, an oral and maxillofacial surgeon, an ENT, a plastic surgeon, a dermatologist, an ophthalmologist, and a psychologist is needed in order to perform the most comprehensive treatment as possible.

Plastic and reconstructive surgery is the base of the treatment. Care of the eyes is of primordial importance. Eye lubricants and antibiotics are used since birth and plastic reconstruction of the eyelids is usually performed with variable results.

Skin needs permanent care with creams, emollients, and oils. Improvement of overall physical appearance has been obtained with botulinum toxin application and acellular dermis grafting. Macrostomia has been treated in several cases by the oral and maxillofacial surgeon. In some cases, the loss of hearing can be improved and the work of a pediatric psychologist is fundamental, before, during, and after the treatment. However, it will not be possible to restore the appearance of this child to a complete normality.

The parents are studying the possibilities, and in the meantime visit to the pediatric psychologist and eye and skin care are suggested. Oral hygiene instruction, oral evaluation, prophylaxis and fluoride application are done periodically.

## 3. Conclusion

This is a new report of an extremely rare condition. In order to arrive to the best option of management and get enough knowledge about rare syndromes, more cases need to be reported. The present case shows, besides the known signs and symptoms, dental manifestations. Enamel hypoplasia is noted in all the primary teeth. To our knowledge, this has not been reported before.

## Figures and Tables

**Table 1:** Clinical manifestations.

Previous cases	Present case
Neurological abnormalities	
Neurological abnormalities	Neurological abnormalities
Cranial deformities	Cranial deformities
Macrostomia	Macrostomia
Absence of eye lashes	Absence of eye lashes
Absence of eyebrows	Absence of eyebrows
Absence or hypoplastic zygomatic arch	Hypoplastic zygomatic arch
Hypertrichosis	
Alopecia	Alopecia
Dry skin	Dry, scaly skin
Folds of skin	
Fingers webbing	
Auricular abnormalities	Auricular abnormalities
Nasal abnormalities	Nasal abnormalities
Genital abnormalities	
Nipple abnormalities	
	Enamel hypoplasia
	Scarring
